# Correspondence

**Published:** 1876-10

**Authors:** G. W. Whitney, W. P. Bemus

**Affiliations:** Jamestown, N. Y.; Jamestown, N. Y.


### Correspondence.

JAMESTOWN, N. Y.

EDITOR BUFFALO MEDICAL AND SURGICAL JOURNAL: The following, if of interest, is at your service:

A case has just presented itself at our office which is so unique that the profession should have the benefit of its record. If such a case has ever been recorded it has escaped our notice or memory.

B. a girl about eight years of age, well developed, health good, has a firm elastic membrane stretched from the uvula to the base of the tongue, firmly and evenly attached throughout the whole circuit. The velum projects about one-fourth the diameter from above. To the left side opposite the point of the velum is an opening about the size of a largs pea. The mother says that she could never swallow fluids but in small quantities and slowly; that solid food could be taken only in very small quantities, and after chewing for a long time. Her voice was very defective and nasal. She was completely relieved by the free use of a eurved pointed bistoury. Hemorrhage slight.

Yours truly,

G. W. WHITNEY, M. D.
W. P. BEMUS, M. D.

